# Supplementary material for: Mental health and psychosocial support strategies in highly contagious emerging disease outbreaks of substantial public concern: A systematic scoping review
Source: PLoS One. 2021 Feb 3;16(2):e0244748. doi: 10.1371/journal.pone.0244748 (PMC7857635; doi:10.1371/journal.pone.0244748)
Supplement: S1 Appendix — Search strategies in three electronic databases. (PDF) [file pone.0244748.s001.pdf]

S1 Appendix. Search strategies. Search strategies in three electronic databases.

MEDLINE OVID

Searched 7 May 2020 [1123 records]

1. Resilience, Psychological/
2. social adjustment/
3. Adaptation, Psychological/
4. (post-traumatic growth or posttraumatic growth or stress-related growth).tw,kf.
5. (positiv$ adj1 (adapt$ or adjust$)).tw,kf.
6. (psychol$ adj1 (adapt$ or adjust$)).tw,kf.
7. (resilien$ or hardiness$).tw,kf.
8. (cope or coping).tw,kf.
9. ((withstand$ or overcom$ or resist$ or recover$ or thriv$ or adapt$ or adjust$ or bounc$ back) adj5 (stress$ or trauma$ or adversit$)).tw,kf.
10. or/1-9
11. exp psychotherapy/
12. Stress, Psychological/th
13. (psychotherap$ or psycho-therap$).tw,kf.
14. (behav$ adj3 (intervention$ or program$ or therap$)).tw,kf.
15. ((cognit$ or cognitive behavior$ or CBT) adj3 (intervention$ or program$ or therap$)).tw,kf.
16. (psycho$ adj3 (intervention$ or program$ or therap$)).tw,kf.
17. relaxation.tw,kf.
18. mindful$.tw,kf.
19. (counsel?ing or coaching).tw,kf.
20. (third wave adj (psycho$ or therap$)).tw,kf.
21. cognit$ restructur$.tw,kf.
22. positive psychology.tw,kf.
23. (refram$ or re-fram$ or reapprais$).tw,kf.
24. (stress adj1 (inoculation or manag$ or reduc$ or resist$)).tw,kf.
25. (anxiety adj3 manage$).tw,kf.
26. "acceptance and commitment".tw,kf.
27. Combined Modality Therapy/
28. (multimodal or multi-modal or combined modal$).tw,kf.
29. exp Health promotion/
30. (health adj3 (educat$ or promot$)).tw,kf.
31. or/11-30
32. 10 and 31
33. (resilien$ adj5 (train$ or program$ or intervention$ or promot$ or prevent$ or enhanc$ or learn$ or teach$ or educat$ or increas$ or develop$ or manag$ or therap$ or protocol$ or treat$)).tw,kf.
34. (hardiness$ adj5 (train$ or program$ or intervention$ or promot$ or prevent$ or enhanc$ or learn$ or teach$ or educat$ or increas$ or develop$ or manag$ or therap$ or protocol$ or treat$)).tw,kf.
35. or/32-34
36. exp Infection/

37. (SARS or influenza or flu or MERS or ebola).tw,kf.
38. Pandemic/
39. pandem$.tw,kf.
40. exp Disease outbreaks/
41. exp Coronavirus/
42. exp Coronavirus infections/
43. (coronavirus or COVID-19 or "2019-nCoV" or "SARS-CoV-2").tw,kf.
44. Quarantine/
45. quarantine.tw,kf.
46. or/36-45
47. 35 and 46
48. exp animals/ not humans.sh.
49. 47 not 48
50. limit 49 to yr="1990 -Current"
51. limit 49 to yr="2000 -Current"

Cochrane Central Register of Controlled Trials (CENTRAL)

Searched 7 May 2020 [2549 records]

#1 [mh "Resilience, Psychological"]
#2 [mh "social adjustment"]
#3 [mh "Adaptation, Psychological"]
#4 ("post-traumatic growth" or "posttraumatic growth" or "stress-related growth")
#5 (positiv* near/1 (adapt* or adjust*))
#6 (psychol* near/1 (adapt* or adjust*))
#7 (resilien* or hardiness*)
#8 (cope or coping)
#9 ((withstand* or overcom* or resist* or recover* or thriv* or adapt* or adjust* or bounc* back) near/5 (stress* or trauma* or adversit*))
#10 {or #1-#9}
#11 [mh psychotherapy]
#12 MeSH descriptor: [Stress, Psychological] explode all trees and with qualifier(s): [therapy - TH]
#13 (psychotherap* or psycho next therap*)
#14 (behav* near/3 (intervention* or program* or therap*))
#15 ((cognit* or cognitive next behavior* or CBT) near/3 (intervention* or program* or therap*))
#16 (psycho* near/3 (intervention* or program* or therap*))
#17 relaxation
#18 mindful*
#19 (counsel*ing or coaching)
#20 (third next wave next (psycho* or therap*))
#21 cognit* next restructur*
#22 positive next psychology
#23 (refram* or re next fram* or reapprais*)
#24 (stress near/1 (inoculation or manag* or reduc* or resist*))
#25 (anxiety near/3 manage*)
#26 "acceptance and commitment"
#27 [mh "Combined Modality Therapy"]

#28 (multimodal* or multi next modal* or combined modal*)
#29 [mh "Health promotion"]
#30 (health near/3 (educat* or promot*))
#31 {or #11-#30}
#32 [mh "Infection"]
#33 (SARS or influenza or flu or MERS or ebola)
#34 [mh "Pandemic"]
#35 pandem*
#36 [mh "Disease Outbreaks"]
#37 [mh "Coronavirus"]
#38 [mh "Coronavirus Infections"]
#39 (coronavirus or COVID-19 or "2019-nCoV" or "SARS-CoV-2")
#40 [mh "Quarantine"]
#41 quarantine
#42 {or #32-#41}
#43 #31 and #42
#44 #31 and #42 with Publication Year from 2000 to 2020, in Trials

Web of Science Core Collection

Searched 7 May 2020 [237 records]

#27 #26
Indexes=SCI-EXPANDED, SSCI, A&HCI, CPCI-S, CPCI-SSH, BKCI-S, BKCI-SSH, ESCI, CCR-EXPANDED, IC Timespan=2000-2020
#26 #24 AND #16 Refined by: WEB OF SCIENCE CATEGORIES: (MULTIDISCIPLINARY SCIENCES OR PUBLIC ENVIRONMENTAL OCCUPATIONAL HEALTH OR SOCIAL SCIENCES BIOMEDICAL OR PSYCHOLOGY MULTIDISCIPLINARY OR PSYCHIATRY)
Indexes=SCI-EXPANDED, SSCI, A&HCI, CPCI-S, CPCI-SSH, BKCI-S, BKCI-SSH, ESCI, CCR-EXPANDED, IC Timespan=1945-2020
#25 #24 AND #16
Indexes=SCI-EXPANDED, SSCI, A&HCI, CPCI-S, CPCI-SSH, BKCI-S, BKCI-SSH, ESCI, CCR-EXPANDED, IC Timespan=1945-2020
#24 #23 OR #20 OR #19 OR #18
Indexes=SCI-EXPANDED, SSCI, A&HCI, CPCI-S, CPCI-SSH, BKCI-S, BKCI-SSH, ESCI, CCR-EXPANDED, IC Timespan=1945-2020
#23 TS=(infect* or SARS OR influenza or flu or MERS or ebola)
Indexes=SCI-EXPANDED, SSCI, A&HCI, CPCI-S, CPCI-SSH, BKCI-S, BKCI-SSH, ESCI, CCR-EXPANDED, IC Timespan=1945-2020
#22 #21 AND #16
Indexes=SCI-EXPANDED, SSCI, A&HCI, CPCI-S, CPCI-SSH, BKCI-S, BKCI-SSH, ESCI, CCR-EXPANDED, IC Timespan=1945-2020
#21 #20 OR #19 OR #18 OR #17
Indexes=SCI-EXPANDED, SSCI, A&HCI, CPCI-S, CPCI-SSH, BKCI-S, BKCI-SSH, ESCI, CCR-EXPANDED, IC Timespan=1945-2020
#20 TS=quarantine
Indexes=SCI-EXPANDED, SSCI, A&HCI, CPCI-S, CPCI-SSH, BKCI-S, BKCI-SSH, ESCI, CCR-EXPANDED, IC Timespan=1945-2020
#19 TS=(coronavirus or COVID-19 or 2019-nCoV or SARS-CoV-2)

Indexes=SCI-EXPANDED, SSCI, A&HCI, CPCI-S, CPCI-SSH, BKCI-S, BKCI-SSH, ESCI, CCR-EXPANDED, IC Timespan=1945-2020
#18 TS=pandem*
Indexes=SCI-EXPANDED, SSCI, A&HCI, CPCI-S, CPCI-SSH, BKCI-S, BKCI-SSH, ESCI, CCR-EXPANDED, IC Timespan=1945-2020
#17 TS=(SARS OR influenza or flu or MERS or ebola)
Indexes=SCI-EXPANDED, SSCI, A&HCI, CPCI-S, CPCI-SSH, BKCI-S, BKCI-SSH, ESCI, CCR-EXPANDED, IC Timespan=1945-2020
# 16 #14 or #15
Indexes=SCI-EXPANDED, SSCI, A&HCI, CPCI-S, CPCI-SSH, BKCI-S, BKCI-SSH, ESCI, CCR-EXPANDED, IC Timespan=1945-2020
# 15 TS=((resilience or hardiness) near/3 (train* or program* or intervention* or promot* or prevent* or enhanc* or learn* or teach* or educat* or increas* or develop* or manag* or therap* or protocol* or treat*))
Indexes=SCI-EXPANDED, SSCI, A&HCI, CPCI-S, CPCI-SSH, BKCI-S, BKCI-SSH, ESCI, CCR-EXPANDED, IC Timespan=1945-2020
# 14 #13 AND #6
Indexes=SCI-EXPANDED, SSCI, A&HCI, CPCI-S, CPCI-SSH, BKCI-S, BKCI-SSH, ESCI, CCR-EXPANDED, IC Timespan=1945-2020
# 13 #12 OR #11 OR #10 OR #9 OR #8 OR #7
Indexes=SCI-EXPANDED, SSCI, A&HCI, CPCI-S, CPCI-SSH, BKCI-S, BKCI-SSH, ESCI, CCR-EXPANDED, IC Timespan=1945-2020
# 12 TS=(health near/3 (educat* or promot*))
Indexes=SCI-EXPANDED, SSCI, A&HCI, CPCI-S, CPCI-SSH, BKCI-S, BKCI-SSH, ESCI, CCR-EXPANDED, IC Timespan=1945-2020
# 11 TS= ((multimodal* or "multi modal*" or "combined modal*") NEAR/3 (treat* or therap* or intervention* or program*))
Indexes=SCI-EXPANDED, SSCI, A&HCI, CPCI-S, CPCI-SSH, BKCI-S, BKCI-SSH, ESCI, CCR-EXPANDED, IC Timespan=1945-2020
# 10 TS=("acceptance and commitment")
Indexes=SCI-EXPANDED, SSCI, A&HCI, CPCI-S, CPCI-SSH, BKCI-S, BKCI-SSH, ESCI, CCR-EXPANDED, IC Timespan=1945-2020
# 9 TS=((anxiety near/1 manag*) or relaxation or mindful* or counsel*ing or coaching or "third wave" or refram* or "re fram*" or "cognitive restructur*" or "positive psychology")
Indexes=SCI-EXPANDED, SSCI, A&HCI, CPCI-S, CPCI-SSH, BKCI-S, BKCI-SSH, ESCI, CCR-EXPANDED, IC Timespan=1945-2020
# 8 TS=(stress near/3 (inoculat* or manag* or reduc* or resist*))
Indexes=SCI-EXPANDED, SSCI, A&HCI, CPCI-S, CPCI-SSH, BKCI-S, BKCI-SSH, ESCI, CCR-EXPANDED, IC Timespan=1945-2020
# 7 TS=((psychotherap* or "psycho therap*") or CBT or mindful* or (behav* near/3 (intervention* or program* or therap*)) OR ((cognit* or "cognitive behavior*" or CBT) near/3 (intervention* or program* or therap*)) OR (psycho* near/3 (intervention* or program* or therap*)))
Indexes=SCI-EXPANDED, SSCI, A&HCI, CPCI-S, CPCI-SSH, BKCI-S, BKCI-SSH, ESCI, CCR-EXPANDED, IC Timespan=1945-2020
# 6 #5 OR #4 OR #3 OR #2 OR #1
Indexes=SCI-EXPANDED, SSCI, A&HCI, CPCI-S, CPCI-SSH, BKCI-S, BKCI-SSH, ESCI, CCR-EXPANDED, IC Timespan=1945-2020
# 5 TS= ((withstand* or overcom* or resist* or recover* or thriv* or adapt* or adjust* or "bounc* back") near/1 (stress* or trauma* or advers*))
Indexes=SCI-EXPANDED, SSCI, A&HCI, CPCI-S, CPCI-SSH, BKCI-S, BKCI-SSH, ESCI,

CCR-EXPANDED, IC Timespan=1945-2020
# 4 TS=(psychol* near/1 (adapt* or adjust*))
Indexes=SCI-EXPANDED, SSCI, A&HCI, CPCI-S, CPCI-SSH, BKCI-S, BKCI-SSH, ESCI, CCR-EXPANDED, IC Timespan=1945-2020
# 3 TS=(positiv* near/1 (adapt* or adjust*))
Indexes=SCI-EXPANDED, SSCI, A&HCI, CPCI-S, CPCI-SSH, BKCI-S, BKCI-SSH, ESCI, CCR-EXPANDED, IC Timespan=1945-2020
# 2 TS=("post traumatic growth" or "posttraumatic growth" or "stress related growth")
Indexes=SCI-EXPANDED, SSCI, A&HCI, CPCI-S, CPCI-SSH, BKCI-S, BKCI-SSH, ESCI, CCR-EXPANDED, IC Timespan=1945-2020
# 1 TS=(resilien* or hardiness*)
Indexes=SCI-EXPANDED, SSCI, A&HCI, CPCI-S, CPCI-SSH, BKCI-S, BKCI-SSH, ESCI, CCR-EXPANDED, IC Timespan=1945-2020
