# Supplementary material for: Mental health and psychosocial support strategies in highly contagious emerging disease outbreaks of substantial public concern: A systematic scoping review
Source: PLoS One. 2021 Feb 3;16(2):e0244748. doi: 10.1371/journal.pone.0244748 (PMC7857635; doi:10.1371/journal.pone.0244748)
Supplement: S1 Table — Detailed eligibility criteria of the systematic scoping review. (PDF) [file pone.0244748.s002.pdf]

S1 Table. Eligibility criteria. Detailed eligibility criteria of the systematic scoping review.

Criterion	Description
**Population**	**Inclusion:**<br>- all ages and sexes; individuals in anticipation of, during or in the aftermath of an outbreak of a highly contagious emerging disease which include COVID-19, and other hazardous infectious diseases (e.g. viral hemorrhagic fevers)<br>- Ebola epidemic outbreak also considered due to fact that Ebola fever belongs to the group of hazardous infectious diseases<br>- irrespective of health status (e.g. healthcare professionals and crisis personnel; children and adolescents; community members; diseased people; people at elevated risk due to somatic conditions)<br>**Exclusion:**<br>- chronic infectious diseases (e.g. HIV/AIDS, tuberculosis, hepatitis B and C, malaria)
**Intervention**	**Inclusion:**<br>- any psychological intervention intended to foster the adjustment (e.g. mental health, resilience, or related constructs) of individuals exposed or possibly exposed to pandemics or another hazardous infectious disease outbreak and/or to promote their mental health and psychosocial support in such situations<br>- focus on improving the participants' ability to cope with the various stressors of a pandemic (e.g. symptoms and death, quarantine)<br>- any setting (e.g. individual or group setting)<br>- any delivery format (e.g. face-to-face or online)<br>- irrespective of content, theoretical foundation (e.g. cognitive-behavioral therapy, mindfulness) and duration<br>- pharmacological (e.g. antidepressant therapy) and physical interventions (e.g. exercise) only if part of psychological training<br>**Exclusion:**<br>- psychological interventions to foster mental health or to promote self-management in people affected by chronic infectious diseases (e.g. HIV)<br>- education interventions to provide knowledge on infectious diseases or to affect behavior change (e.g. hygiene education)<br>- interventions aimed at increasing vaccination rates or at improving immune responses to influenza vaccine<br>- psychological interventions to prevent or to reduce the symptom severity of acute respiratory infections (e.g. flu symptoms)<br>- experimental studies to test the effect of health communication manipulation

**Comparison**	No eligibility criteria
**Outcome**	No eligibility criteria
**Study design**	**Inclusion:**<br>- quantitative and qualitative studies focusing on or measuring the effects of the above-defined intervention as well as mixed-methods research designs (e.g. individual and cluster-RCTs, randomized studies, single-group studies, case studies or reports) in order to gain comprehensive view of existing interventions and the body of corresponding research<br>**Exclusion:**<br>/
**Publication date**	No restrictions
**Publication language**	No restrictions
**Publication format**	No restrictions
